# Digital Platform Uses for Help and Support Seeking of Parents With Children Affected by Disabilities: Scoping Review

**DOI:** 10.2196/37972

**Published:** 2022-12-06

**Authors:** Oliver Gruebner, Afua van Haasteren, Anna Hug, Suzanne Elayan, Martin Sykora, Emiliano Albanese, John Naslund, Markus Wolf, Marta Fadda, Michael von Rhein

**Affiliations:** 1 Department of Geography University of Zurich Zurich Switzerland; 2 Epidemiology, Biostatistics, and Prevention Institute University of Zurich Zurich Switzerland; 3 Institute of Public Health Università della Svizzera italiana Lugano Switzerland; 4 Centre for Information Management Loughborough University Loughborough United Kingdom; 5 Department of Global Health and Social Medicine Harvard Medical School Boston, MA United States; 6 Department of Psychology University of Zurich Zurich Switzerland; 7 University Children’s Hospital Zurich Zurich Switzerland

**Keywords:** digital place, pediatric diagnoses, conditions, disability, neuromuscular, information and support seeking, online, social media, peer support, lived experience, parents, children, youth, review, scoping review, trauma, caregivers

## Abstract

**Background:**

Receiving a diagnosis that leads to severe disability in childhood can cause a traumatic experience with long-lasting emotional stress for patients and family members. In recent decades, emerging digital technologies have transformed how patients or caregivers of persons with disabilities manage their health conditions. As a result, information (eg, on treatment and resources) has become widely available to patients and their families. Parents and other caregivers can use digital platforms such as websites or social media to derive social support, usually from other patients and caregivers who share their lived experiences, challenges, and successes on these platforms. However, gaps remain in our understanding of platforms that are most frequently used or preferred among parents and caregivers of children with disabilities. In particular, it is not clear what factors primarily drive or discourage engagement with these digital tools and what the main ethical considerations are in relation to these tools.

**Objective:**

We aimed to (1) identify prominent digital platforms used by parents or caregivers of children with disabilities; (2) explore the theoretical contexts and reasons for digital platform use, as well as the experiences made with using these platforms reported in the included studies; and (3) identify any privacy and ethical concerns emerging in the available literature in relation to the use of these platforms.

**Methods:**

We conducted a scoping review of 5 academic databases of English-language articles published within the last 10 years for diseases with childhood onset disability and self-help or parent/caregiver-led digital platforms.

**Results:**

We identified 17 papers in which digital platforms used by parents of affected children predominantly included social media elements but also search engines, health-related apps, and medical websites. Information retrieval and social support were the main reasons for their utilization. Nearly all studies were exploratory and applied either quantitative, qualitative, or mixed methods. The main ethical concerns for digital platform users included hampered access due to language barriers, privacy issues, and perceived suboptimal advice (eg, due to missing empathy of medical professionals). Older and non–college-educated individuals and ethnic minorities appeared less likely to access information online.

**Conclusions:**

This review showed that limited scientifically sound knowledge exists on digital platform use and needs in the context of disabling conditions in children, as the evidence consists mostly of exploratory studies. We could highlight that affected families seek information and support from digital platforms, as health care systems seem to be insufficient for satisfying knowledge and support needs through traditional channels.

## Introduction

Receiving the diagnosis of a disease leading to disability in childhood can cause long-lasting emotional stress for patients and family members. There is considerable evidence illustrating the adverse mental health consequences and higher levels of psychological distress experienced among children with disabilities [1,2]. Similarly, parents and caregivers (hereafter referred to as “parents” for simplification) of children with disabilities also experience elevated stress and can face challenges adapting to the care needs of their child [3]. Importantly, efforts are needed to support parents in adapting and meeting the needs of their child, as this can directly impact the child’s development and well-being over the life course [4,5]. Therefore, it is critically important to determine effective approaches for supporting parents of children with disabilities, so that they can adopt necessary and desired coping strategies and feel confident in meeting the day-to-day needs of their children.

In recent decades, emerging digital technologies have transformed how patients or parents of persons with disabilities manage their health conditions [6], and information (eg, on treatment and resources) has become more widely available to them. Furthermore, social and emotional support (eg, through online self-help and peer support groups) is now more readily accessible through various online platforms. For instance, Oldenburg et al [7] present a helpful rundown of the role new media have played (eg, PatientsLikeMe) in supporting patients of children with chronic diseases, while in the work of Sykora [8], some early health-related social platforms are mentioned (eg, PatientOpinion, CarePages, CureTogether, and PatientsLikeMe), and a walkthrough of the social platform CureTogether (now defunct after being bought by 23andMe) is provided. Most recently, patients suffering from “long COVID” (referring to the recent COVID-19 pandemic) who were being dismissed by their health care professionals were able to mobilize by sharing their symptoms and locating other sufferers through social media. This ultimately resulted in a new chronic condition known as “long COVID” and the creation of what are now known as “long COVID clinics” to support patients [9]. Parents of children with debilitating diseases can potentially use digital platforms such as search websites or social media (eg, Reddit or WhatsApp groups) to derive social support, usually from other patients and parents who share their lived experiences, challenges, and successes on these platforms.

However, gaps remain in our understanding of platforms that are most frequently used or preferred among parents of children with disabling conditions. For example, it is not clear what factors drive or discourage engagement with these digital tools. In addition, there is little evidence available about the ethical concerns over services provided by digital platforms that are used by parents for information and support seeking in the context of a disabling or lethal disease of their child.

Accordingly, in this scoping literature review, we aimed to (1) identify prominent digital platforms used by parents or caregivers of children with disabilities; (2) explore the theoretical contexts and reasons for digital platform use, as well as experiences with using these platforms reported in the included studies; and (3) identify any privacy and ethical concerns emerging in the available literature in relation to the use of these platforms.

## Methods

### Study Design

We conducted a scoping review following the framework of Arksey and O’Malley [10]. Scoping reviews are useful in mapping and identifying available evidence [11]; therefore, we opted for this approach rather than other types of reviews, which often answer a single clinical question, because we were more concerned with broadly exploring a concept [12]. The search was performed using 5 scientific databases: PubMed, CINAHL, PsycINFO, Communication & Mass Media Complete, and Psychology & Behavioral Sciences Collection. EBSCO Host was used to concurrently search through all the databases except for PubMed. In line with Arksey and O’Malley [10], the reference lists of the articles included were screened for additional studies. Gray literature searches were also conducted on the websites of various major organizations tackling neuromuscular diseases (NMDs; Multimedia Appendix 1), in addition to using Google search engine to retrieve further studies. All the searches were conducted between July and September 2021.

### Search Strategy: Identifying Relevant Studies

Based on the severity of the disease and the high psychoemotional distress it can cause to the parents of affected children, initial searches began with a primary focus on retrieving studies relating to NMDs with a pediatric onset such as Duchenne muscular dystrophy (DMD). However, these searches resulted in few studies relevant to the subject of interest. Therefore, a search strategy was adopted to include “disabilities” as a broader keyword. Table 1 details the keywords and search terms used to identify relevant studies. The inclusion criteria to identify relevant papers were (1) scientific English articles published in the last 10 years (2011- 2021) on diseases with childhood-onset disability, (2) all study types (eg, reviews, original studies), (3) use of self-help or parent/caregiver-led digital platforms (eg, internet, websites, social media or online support groups), and (4) those describing either reasons, expectations, concerns, suggestions, or experience on digital platforms. The exclusion criteria were (1) non-English articles, (2) articles published before 2011, (3) adult-onset diseases, (4) papers reporting on digital platforms maintained by medical institutions or those designed for research, and (5) papers with a main focus on health professionals’ experiences with digital platforms.

The exclusion of non-English articles was due to our inability to analyze articles in non-English languages at the time of our research; however, we must emphasize that we will endeavor to include other languages in future studies. Our focus on the past decade in our inclusion criteria is due to the relatively recent emergence of social media, which only appeared in the first decade of the 21st century and gained increasing popularity [13] in its current form from around 2009 onward. The landscape and nature of social media’s interactive affordances have also evolved substantially [14], which is why we deemed that extending the study period beyond 1 decade would become problematic.

Two authors (AH and AvH) independently screened the titles, abstracts, and full texts, while a third author (MF) was consulted to establish a consensus.

**Table 1:** Keyword searches conducted on titles and abstracts.

Key concepts	Search terms
Parents/caregivers	parent* OR caregiver* OR carer* OR mother* OR father* AND
Children affected by disability	‘child* disab*’ OR ‘child* disorder*’ OR ‘pediatric disab*’ OR ‘disabled persons’ OR ‘physical disab*’AND
Communication, exchange	communicat* OR experienc* OR challenge* OR connect* OR support* OR exchang* AND
Internet/social media support	Internet* OR online* OR ‘social media*’ OR webs* OR virtual* OR ‘online support’ OR ‘Self-help Groups’ OR Facebook OR Twitter OR WhatsApp OR Reddit OR Instagram OR ‘mobile App*’

### Charting the Data

The descriptive attributes of each article including the authors, year of publication, country, and objective of the study were extracted from each article. To facilitate the process of identifying the most prolific digital platforms, the scope of each study and its objectives, along with the respective outcomes measured, were also extracted from each full-text article included in the review.

### Collating, Summarizing, and Reporting the Results

To derive an overview of the informational needs of parents of children with disabilities, the preferred online platforms, specific experiences, expectations, concerns, and suggestions for improvement highlighted by each study were identified. These were later labeled under broader concepts and organized around more general, coherent themes. In the last stage of the analysis process, common and divergent themes and topics in findings among and across all the included articles were identified.

## Results

### Initial Findings

**Figure 1:**
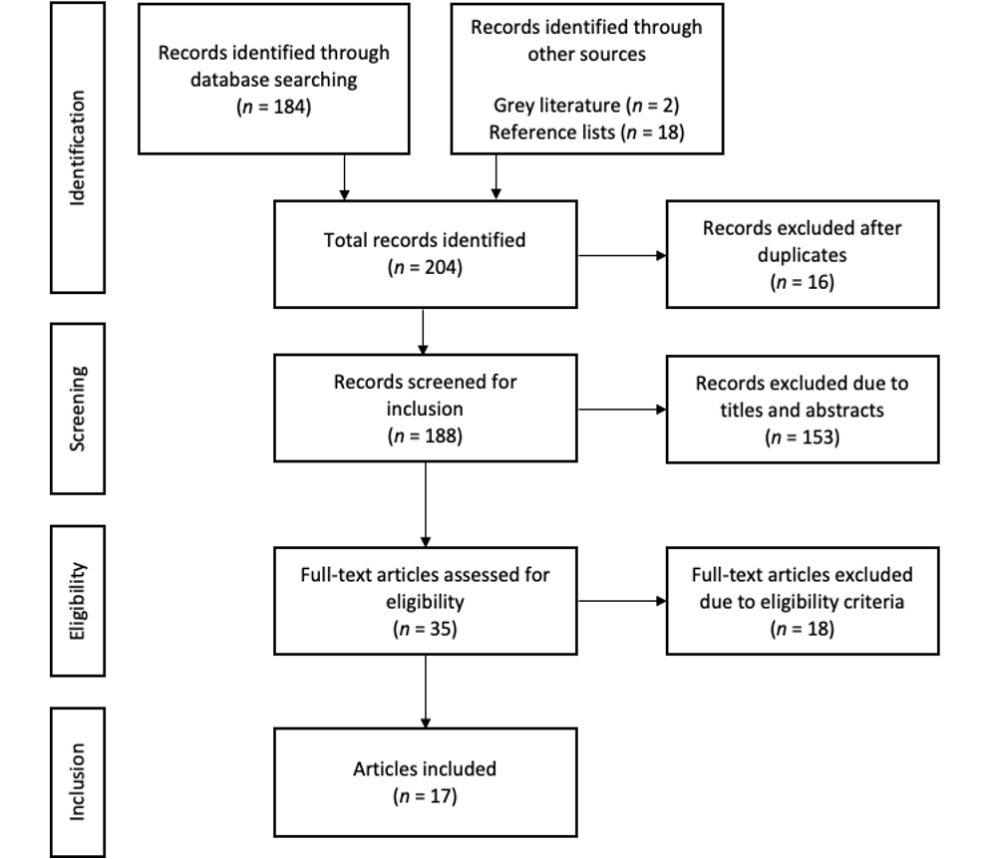
PRISMA (Preferred Reporting Items for Systematic Reviews and Meta-analyses) flowchart.

Our search yielded a total of 184 scientific articles. Additionally, 18 articles were identified by reference list screenings, and 2 articles [15,16] were obtained from gray literature, bringing the total number of retrieved articles to 204. Of these, 16 records (7.8%) were identified as duplicates and excluded. Of the resulting 188 articles (100%), 153 (81.4%) were excluded according to the inclusion and exclusion criteria based on their titles and abstracts. Subsequently, we screened the full texts of the remaining 35 articles (100%) and further excluded 18 articles (51.4%) based on our inclusion and exclusion criteria, with 17 final articles (100%) included in the review. The PRISMA (Preferred Reporting Items for Systematic Reviews and Meta-analyses) flow diagram in Figure 1 displays the entire process involved in selecting the included papers.

Detailed information about the year of publication, study design and sample size, location, study objective, study population, main digital platform, and outcomes measured can be found in Table 2. The earliest study included in our review was conducted in 2011, while the most recent study was conducted in 2020 [15,17]. Most studies (n=14, 82.4%) were original and observational, applying either qualitative (n=5, 29.4%) [15,18-21], quantitative (n=5, 29.4%) [17,22-25], or mixed methods approaches (n=4, 23.5%) [26-29]. The remaining articles consisted of 2 (11.8%) reviews [30,31] and 1 (5.9%) case study [16]. Mothers made the bulk of the study participants in all the included studies aside from Ammari and Schoenebeck [20], where efforts were made to overrecruit fathers. In 1 (5.9%) study (Rocha and colleagues [24]), the gender of parents was not identified, likely due to the study’s recruitment of participants through 2 online registries (Simons Variation in Individuals Project and GenomeConnect). The target population in the studies was most commonly defined as parents of children across a range of disorders and special needs (n=13, 76.5%), while 1 (5.9%) study’s population focused on families in general [26], 2 (11.8%) on patients themselves [24,25], and 1 (5.9%) solely on married mothers with up to 5 children [19]. Most studies were conducted in the United States (n=5, 29.4%) [15-17,19,29], while participants for 6 studies (35.3%) were derived from multiple countries through online recruitment [20,23,24,27,30,31]. The remaining studies were from Australia (11.8%) [26,28], Italy (5.9%) [25], Kuwait (5.9%) [22], Norway (5.9%) [21], and the Netherlands (5.9%) [18].

**Table 2:** Data extracted from the included articles.

Author, year	Study design (N)	Location	Study objective	Study population	Main digital platform^a^ (outcome measured^b^)
Gundersen, 2011 [21]	Interviews (N=10)	Norway	Internet use for coping with chronic illness resulting from rare genetic disorders	Parents whose children have rare genetic disorders	1, 2 (a, b, c, e)
Knapp et al, 2011 [17]	Survey (N=2371)	United States	Low-income parents of children with special needs access and use; factors related to internet use; parents’ eHealth literacy, and factors associated with higher eHealth literacy	Parents of children with special health care needs	1, 2 (a, d, e)
Tozzi et al, 2013 [25]	Survey (N=516)	Italy	Details internet user profiles and how internet use affects decision-making	Patients of rare diseases	1, 2 (a, c, e)
Johnston et al, 2013 [26]	Mixed methods: survey, (N=522), focus group (N=21)	Australia	How the internet can assist families with young disabled children to make effective intervention and support decisions	Families of young children with disabilities	1, 2 (a, d, e)
Ahmed, 2014 [31]	Literature review (N=15)	Online	Summarize existing recommendations on internet use by parents of children with rare and difficult illnesses	Parents whose children have rare, difficult illnesses and special needs	1, 2 (c, d)
Ammari et al, 2014 [29]	Mixed methods: interview (N=18), survey (N=205)	United States	Use of social media sites by parents of children with special needs for information and social support; perception and management of online and offline judgment; posts perceived to be socially appropriate to post on their own online profiles versus in shared online groups; how social media sites can better support special needs families	Parents of children with special needs	1 (a, c, d)
Al-Daihani and Al-Ateeqi, 2015 [22]	Survey (N=240)	Kuwait	Information seeking behavior of parents of children with disabilities	Parents of children in a school for special needs	1, 2 (a, c, e)
Ammari and Schoenebeck, 2015 [20]	Semistructured interviews (N=43)	Online	The use of social media needs by parents with special needs children	Parents of children with special needs	1 (a, c, e)
Russell et al, 2016 [23]	Quantitative assessment of Facebook likes and posts; survey (N=49)	Canada, United Kingdom, Australia	Development and evaluation of web-based research advisory community that links parents to researchers to improve research and affected families/children’s lives	Parents of children with special needs who used a Facebook group	1 (a, b, c, d)
DeHoff et al, 2016 [30]	Scoping review (N=N/A^c^), expert interviews (N=N/A)	Online	Status of research on the usefulness of digital communication like social media, in providing informational and emotional support	Parents of young children with special health care needs	1, 2 (a, b, c, e)
Fostervold, 2016 [16]	Case study (N=1)	United States	How social media posts support parents in raising their children with a disability	Parents of a child with a disability	1 (a)
Alsem et al, 2017 [18]	Semi-structured interviews (15)	Netherlands	Information needs, process of seeking and evaluating information, and the different sources of information for parents	Parents of children with disabilities	1, 2 (a, b)
Nicholl et al, 2017 [27]	Mixed methods: survey (N=128), focus group, (N=8)	Ireland, Northern Ireland, United States, United Kingdom	General internet usage patterns, types of information frequently searched for, and effect of internet-sourced information on parents of children with rare conditions	Parents of children with rare conditions	1, 2 (a, b, d)
Sharaievska and Burk, 2018 [19]	Semistructured interviews (N=8)	United States	Role of online and offline support groups in the lives of families with children who have developmental disabilities	Married mothers who had 1-5 children with developmental disabilities	1 (a, e)
Rocha et al, 2018 [24]	Survey (N=103)	Online	Understand the online behavior, perspectives, and norms of rare disease communities to provide preliminary guidance to genetic counselors who wish to have discussions about social media support resources	Patients with newly described or rare genetic findings from online patient registries	1 (a, c, d)
Tracey et al, 2018 [28]	Mixed methods: survey (N=291), focus group (N=56)	Australia	Information-seeking behavior of parents and their perceptions and evaluations of the various information sources available	Parents of children with disabilities	1, 2 (a, b, c, d, e)
Terra, 2020 [15]	Semistructured and open-ended interviews (N=5)	United States	Role of social media to empower and provide community for parents raising children with profound multiple disabilities	Parents of children with profound multiple disabilities	1 (a, c)

^a^Digital platforms: (1) social media (eg, Facebook, Twitter, email), (2) internet search engines, health apps, medical websites, or not specifically mentioned otherwise.

^b^Outcome measured: (a) reasons for use, (b) expectations from use, (c) concerns/shortcomings, (d) suggestions for improvement, (e) satisfaction and experience.

^c^N/A: not available.

### Digital Platforms Utilized

We classified the types of digital platforms identified in the reviewed articles into 2 categories: (1) digital platforms with social interaction options, such as social media; and (2) other platforms, such as search engines, medical websites, and health-related apps. Due to the overall aim of this review and the search strategy applied, health-related apps were not prominently found. As listed in Textbox 1, social media were the most prolific digital platforms used by caregivers and parents and were mentioned in 3 (17.7%) of 17 papers [17,21,22]. Furthermore, 1 (5.9%) study [19] focused entirely on online support groups by comparing the differences between online and offline interactions, whereas all other studies (94.1%) examined online support within the context of other digital platforms [21-26]. Some studies (n=4, 23.5%) reported on the use of internet search engines [18,25,27,28] or other online information sources (n=2, 11.8%) [17,26]. Medical websites that were frequented by caregivers were also identified in some studies (n=4, 23.5%) [21,22,25,29]. Differences in digital platform preference were evident among different age groups, as noted by Tozzi and colleagues [25]. They found that compared to younger age groups, respondents 55 years or older appeared to be less familiar with Twitter or smartphones, preferring to use email and Facebook instead [25].

**Digital platforms mentioned in the reviewed literature.**

1. Platforms with social interaction options, such as Facebook, Twitter, and Instagram, YouTube, Pinterest, LinkedIn, Skype, Viber, MSN messenger, Yahoo! Answers (operating between June 2005 and May 2021), Yahoo Groups, Quora, Google groups, CaringBridge, CarePages (shut down in December 2017), and other online forums, blogs, discussion boards, and emails
2. Other platforms, such as search engines, medical websites (BabyCenter website, Better Start website, autism support websites), and health-related apps

### Theoretical Contexts and Reasons for Digital Platform Use and Experiences Made

Overall, 5 (29.4%) studies [15,16,19-21] adopted various theoretical frameworks guiding the understanding of how social interactions and support work. First, the Ecological Model of Human Development, as used in Fostervold [16], is a theory that helps us understand the interconnectedness of family and the larger society and the resulting socialization of a child. The Symbolic Interaction Framework [32] employed in the study by Sharaievska and Burk [19] suggests that individuals’ perception of reality is constructed through their interaction with the people and objects around them. Terra’s thesis [15] applied 2 theories, namely, the Theory of Sense of Community and the Empowerment Theory. Based on the Theory of Sense of Community developed in 1976 and published in 1986 by McMillan and Chavis [33], this thesis “sought to explain the dynamics of the sense-of-community force” [15]. The identified components of sense of community were membership, influence, fulfillment of needs, and shared emotional connection [15]. The Empowerment Theory describes a process in which people gain understanding and control over personal, social, economic, or political forces in order to take action to better their lives, and it was utilized in the study by Terra [15] to focus on the impact of community membership on education, awareness, and action on behalf of their child and other children with disability. Another study [20] also focused on the Empowerment Theory and extended it into a new theory of “networked empowerment” that describes how parents whose children have received a special needs diagnosis find other parents, mobilize resources, and become advocates. The fifth study [21] used the theoretical framework of medical sociologist Aaron Antonovsky [34-36], who was dedicated to understanding how people manage to demonstrate resilience despite going through extremely difficult life experiences. Antonovsky contends that the explanation is to be found in people’s capacity to manage stressors, that is, “demands to which there are no readily available or automatic adaptive responses” [36].

From the reviewed literature, we noted that digital platforms were predominantly used for information retrieval and social support. As noted by Gunderson [21], no 2 digital platforms were considered equivalent for deriving various types of information by their study participants. Therefore, parents chose to use either platform based on their respective needs. The criteria considered necessary to facilitate the utility of platforms were highlighted in 2 studies. According to Nicholl et al [27], the most important attributes of platforms were relevance, accurate and up-to-date information, trustworthiness, recommendation by health professionals, easy-to-understand information, helpful references, and an appealing layout. Participants in Johnston and colleagues’ [26] study echoed several of these factors, adding that presentation (different languages, videos or audio recordings, pictures, easy to navigate, and information written in easy language) and connection functionality (blog, forum, access to professionals and other parents, and access to owners of the website) increased the overall utility of a platform.

The general expectation that digital platforms would have objective, up-to-date, and vital information on conditions of interest was emphasized by participants in several other studies [18,20,22-24]. The types of information sought by parents included details about services and systems available [29,30], specialists for specific conditions [20,25], social workers [20], and appropriate schools and childcare [23]. Parents used these types of information to assist them in caring for their children as well as interacting with professionals involved in their care. They often felt empowered by the readily available information on digital platforms. In several studies, parents particularly felt the need to consult digital platforms soon after a diagnosis to learn more about the condition or before an upcoming doctor’s visit [18,20-22,27].

Digital platforms also provided a means of not only communicating with parents familiar with the condition of interest but also scheduling appointments with professionals, seeking second opinions or alternative therapies [25], or communicating with family and friends [27]. For example, parents used websites such as CaringBridge and CarePages to provide updates on the status of their children’s health [29]. Digital platforms such as CaringBridge and CarePages offer the opportunity to post about the status of one’s condition with the primary aim of assisting others frequenting these platforms. Some parents chose to share relevant scientific research on the condition faced by their children for the benefit of others, especially after gaining more experience with services and diagnoses [16].

Participants in several studies stated that digital platforms would foster a feeling of support among the participants [18,20,22-24]. By consulting the posts by parents of children with similar symptoms and care pathways, most parents became more attenuated to what to expect and how best to care for their children [25]. Moreover, some participants in a study by Ammari and Schoenebeck [20] noted that posts from other parents (eg, on health care services and medication, special education services, or specially designed clothes) provided hope and decreased their anxiety and depression after a diagnosis. Several studies reported that parent-to-parent peer support either via social media groups or online support groups was vital in reducing feelings of isolation among parents of children with special needs [15,16,19,24]. The same was true for respondents in Gunderson’s [21] study, who reported that sole help from health professionals proved insufficient, especially after initial diagnosis or during the deterioration phase of a condition. Where professionals or researchers participate in forums on digital platforms, respondents stressed the importance of their posts reflecting empathy [23]. In addition, humor was considered a viable tool to minimize the emotional toll of social media posts, according to participants in the study by Ammari and colleagues [29].

Although digital platforms were preferred in most instances because virtual interactions were easier to establish and manage, some parents hoped for the development of hybrid social connections whereby virtual relationships would translate into occasional physical interactions [15]. In other studies [20,29], online interactions through social media sites were reported to facilitate social support, especially for geographically restricted families with scarce resources in their immediate vicinities. However, social media sites were also reported to not be facilitative in linking newly diagnosed individuals and their families with experienced ones or connecting affected individuals to others with analogous experiences [29].

### Privacy and Ethical Concerns in the Use of Digital Platforms

When using social media in the context of child disability, privacy issues were imminent among several parents, as personal posts relating to photos and medical questions, for instance, were often restricted to closed groups [23,24,28]. Some studies showed that the majority of participants preferred closed over open online fora, such as closed Facebook groups to discuss personal information only with members of the group [18,20,24]. Furthermore, closed Facebook pages were preferred by participants in the study by Ammari and Schoenebeck [20] for organizing and strategizing activities, whereas public groups were used to advocate for perceived necessary policy changes. While 1 study found that the number of respondents feeling rather or very comfortable with sharing medical and personal information in a closed group decreased when having professionals present [24], there was a consensus in opinion about the presence of professionals on digital platforms, as they were considered necessary by some parents to facilitate robust information sharing [19,24,26,29].

According to Fostervold [16], issues of conflict of interest and privacy also arise when participants request to be “friends” with their health professionals on social media websites. Furthermore, possible abuse of photographs of children and medical information was noted by 1 participant in the study by Rocha et al [24]. Although parents reported feeling overall less judged online than offline, they dealt with judgment online by blocking or unfriending culprits, minimizing posts, reducing their engagement, and even deleting the respective digital platform account [29].

We also found that there were differences in digital platform use according to the sociodemographics involved. For example, the study by Tozzi and colleagues [25] found that individuals who were younger, active on social media, and already prone to communicating via electronic means were the most likely to discuss information found online with physicians. Conversely, the study by Knapp and colleagues [17] found that older individuals, non–college-educated people, non-English speaking people, and ethnic minorities were less likely to access information online [17]. The same study also found that these population groups, when compared to their reference group, were less likely to show eHealth literacy based on the eHealth Literacy Scale (eHEALS), a measure to evaluate the “ability to locate, evaluate, integrate, and apply information gained from electronic platforms” [17,37]. The language barrier of digital platforms also prevented many parents from interacting with and deriving optimum utility from digital platforms [26,28].

Digital platforms on which information was obscured and difficult to find also posed a great concern for participants [18]. Additionally, the prevalence and traction of misinformation and disinformation on digital platforms were considered particularly problematic among participants of 2 studies [15,24]. Furthermore, the expectation for unrealistic lifestyles [15,29], along with depressing posts [15,20,21,25], posed a mental health worry. For some parents, the difficulty of weighing advice found on social media information against that of professionals [15,25] was also an issue of concern. Whereas posts linked to government sources were deemed important to increase the trustworthiness of information in some studies [18], other studies found this to be insufficient and advocated for posts to include information on the original cultural context [28].

Suggestions made in another study to increase the usefulness of social media platforms included targeted pages to connect children with similar ages and conditions together, consolidating pages on similar conditions, and facilitating the online interaction between more disease-experienced parents with less experienced ones [29]. Finally, health apps focused on delivering interventions were encouraged to include and prioritize social support elements to improve their overall utility [30].

## Discussion

### Principal Results

The available literature shows that digital platforms used by parents of children with disabilities predominantly included social media but also search engines, health-related apps, and medical websites. Information retrieval and social support seeking were the main reasons for their utilization, with the general expectation of finding and sharing objective, up-to-date, and reliable information and guidance. In addition, the main concerns for digital platform users included privacy issues and the digital divide across sociodemographic groups, including language barriers.

### Social Support From Digital Places

In our review, most of the literature reported that parents used commonly available social media platforms (eg, Facebook, Twitter, Instagram) and other online forums, blogs, and discussion boards. Social media can be defined as digital platforms that provide users with the ability to share and discuss information publicly and within individual peer networks [38]. As such, they offer a social component that includes bidirectional communication among social media users that allows for social interaction and exchange. In previous studies, researchers recognized social media platforms as so-called “digital places” that can be defined as socially constructed spaces (ie, environments) with individual meaning and utility to their users, similar to geographic places [38,39]. Following the nomenclature of Glanz et al [40], respondents in our reviewed studies used these digital places for informational and emotional support. It is noteworthy that some parents felt less charged online than offline, possibly due to the virtual character of digital places and more options to defriend or retract from social contacts more easily than in the physical world. Participants in the reviewed studies expressed the general expectation to find and share objective, up-to-date, and reliable information and guidance, which seems to be closely related to a feeling of empowerment. Importantly, this need for information seems to be closely related to the need for emotional and other forms of social support. Future research may extend the focus on multiple dimensions of digital places—how individual meaning and utility of these places may influence their use in the context of disabilities in children. Furthermore, future research should also investigate the question of how digital place use in this context might affect mental health and resilience in patients and family members, especially during the time of diagnosis and at critical events during disease progression.

### Ethical and Privacy Concerns Reported in Digital Platform Use

Individual-level characteristics and social determinants of health played a role in digital platform use. For example, older and less educated individuals, as well as ethnic minorities, were less likely to access information online compared to their younger, college-educated, White counterparts. This finding lends itself to the explanation that online digital platforms and resources are not easily accessible to everyone and once more indicates a digital divide in the context of child disability, with less access for already vulnerable families. Our review also highlighted that women were the respondents in most of the reviewed studies; this may point to a gender bias, but it may also suggest that women take over the larger care burden in families with children living with a disability. However, it is worth noting that the results on sociodemographics and digital platform use were rather old, in that they were published in 2013 [25] and 2011 [17], when smartphones were not as widely used. According to the Pew Research Center, 53% of adults in the United States owned a smartphone in 2013 [41], and 85% owned a smartphone in 2021 [42]. Nevertheless, our findings from these 2 references highlight a prevalent issue where older adults are often less likely to be familiar with the most recent social media platforms.

From a geographic perspective, the reviewed studies derive from many different countries, and it is unclear whether there are patterns of digital platform use that are distinct in some regions or others. Some of the platforms might be specifically useful or even targeted to regional, national, or cultural audiences, which should be investigated in future studies.

Privacy issues were raised in various studies, highlighting that affected individuals and parents felt more confident in closed fora and that they appreciated if professionals were verifying the information being discussed. At the same time, there was a desire to try to maintain a healthy distance from professionals to discuss private issues in a safe space. This finding points to the ambivalent relationship that parents of children with disabilities may develop with the child’s health care providers.

No study in our review applied an experimental design involving the evaluation of digital platforms to test for the effects of distinct platform designs on distinct dimensions of support (ie, emotional, informational, and instrumental support and appraisal). This represents a significant limitation in the available literature because it means limited evidence in this area, as well as difficult-to-draw conclusions about the effectiveness of these platforms beyond anecdotal accounts from the explorative research summarized in this review. This is particularly true for patients with NMDs on whom research in this field seems to be widely neglected thus far despite the severity of the diseases.

Our findings have major practical implications. Physicians and other health care providers, health care facilities, and health agencies should take advantage of digital platforms that provide social interaction options to meet and empower families of patients living with disabilities. This should be done by not only identifying and addressing patients’ and parents’ needs before, during, and after access but also by recognizing and correcting any structural conditions that may affect individuals’ opportunities to use such platforms.

### Limitations

Our review is biased toward high-income countries; therefore, the relevance of the findings for use across different settings globally is difficult to ascertain. Future studies should address underrepresented cultural groups, languages, races, ethnicities, and countries to broaden our understanding of social media use in the context of pediatric diagnoses leading to disabilities and the inequities associated with it.

### Conclusions

To date, scarce scientifically sound knowledge is available on digital platform use and needs in the context of disabling diagnoses in children. Our study aims to help fill this gap by highlighting which digital platforms families of children with disabilities visit, what they seek in them, and why. Most importantly, our findings on the privacy and ethical concerns in the use of these platforms remind us of the role of social determinants in shaping the magnitude of individuals’ access to and benefit from these platforms. As families of children with disabilities constitute an already vulnerable population, future research should seek to identify and critically examine the avoidable, unfair, and unjust conditions that may amplify forms of inequities in their access to support. This can be done by continually committing to engage a broad range of narratives, voices, and lived experiences when conducting empirical research on digital platform uses among parents of children affected by disabilities.

## Appendix

Multimedia Appendix 1 Patient association websites.

## Abbreviations

DMD: Duchenne muscular dystrophy
eHEALS: eHealth Literacy Scale
NMD: neuromuscular disease
PRISMA: Preferred Reporting Items for Systematic Reviews and Meta-analyses
