# Supplementary material for: Digital Platform Uses for Help and Support Seeking of Parents With Children Affected by Disabilities: Scoping Review
Source: J Med Internet Res. 2022 Dec 6;24(12):e37972. doi: 10.2196/37972 (PMC9768636; doi:10.2196/37972)
Supplement: Multimedia Appendix 1 [file jmir_v24i12e37972_app1.docx]

## Appendix 1. Patient association websites.

| **Patient Association websites**  AANEM: <https://www.aanem.org/Home>  Australasian Neuromuscular Disease Network: <https://www.ann.org.au>  Duchenne Australia: <https://www.duchenneaustralia.org>  European Alliance of Neuromuscular Disorders Associations: <http://www.eamda.eu>  FSRMM: <https://www.fsrmm.ch/home>  German Muscular Society: <https://www.dgm.org>  Muscular Dystrophy Association: <https://www.mda.org>  Muscular Dystrophy Canada: <https://muscle.ca>  Muscular Dystrophy UK: <https://www.musculardystrophyuk.org>  Neuromuscular Disease Foundation: [www.curehibm.org](http://www.curehibm.org)  Neuromuscular Disease Support Organizations: <https://neurology.ufl.edu/divisions/neuromuscular/neuromuscular-support-organizations/>  NMD4C: <https://neuromuscularnetwork.ca>  SMA Europe: <https://www.sma-europe.eu>  Swiss Muscular Society: <https://www.muskelgesellschaft.ch>  Swiss Duchenne Foundation: <https://progena.ch/en/>  Treat NMD: <https://treat-nmd.org> |
| --- |
